# Tipping points emerge from weak mutualism in metacommunities

**DOI:** 10.1371/journal.pcbi.1011899

**Published:** 2024-03-05

**Authors:** Jonas Denk, Oskar Hallatschek

**Affiliations:** 1 Department of Physics, University of California, Berkeley, California, United States of America; 2 Department of Integrative Biology, University of California, Berkeley, California, United States of America; 3 Peter Debye Institute for Soft Matter Physics, Leipzig University, Leipzig, Germany; Abdus Salam International Centre for Theoretical Physics, ITALY

## Abstract

The coexistence of obligate mutualists is often precariously close to tipping points where small environmental changes can drive catastrophic shifts in species composition. For example, microbial ecosystems can collapse by the decline of a strain that provides an essential resource on which other strains cross-feed. Here, we show that tipping points, ecosystem collapse, bistability and hysteresis arise even with very weak (non-obligate) mutualism provided the population is spatially structured. Based on numeric solutions of a metacommunity model and mean-field analyses, we demonstrate that weak mutualism lowers the minimal dispersal rate necessary to avoid stochastic extinction, while species need to overcome a mean threshold density to survive in this low dispersal rate regime. Our results allow us to make numerous predictions for mutualistic metacommunities regarding tipping points, hysteresis effects, and recovery from external perturbations, and let us draw general conclusions for ecosystems even with random, not necessarily mutualistic, interactions and systems with density-dependent dispersal rather than direct mutualistic interactions.

## Author summary

In ecosystems with obligate mutualism, species rely on each other’s cooperation to thrive. Obligate mutualism has been of special interest in theoretical ecology because it generates tipping points between drastically different ecological states (along with bistability). Weak, non-obligate mutualistic interactions have attracted much less interest due to their minimal impact on population growth behavior in well-mixed scenarios. However, in spatially structured metacommunities with migration coupling and demographic stochasticity, we find that weak mutualism can fundamentally alter population growth behavior, leading to bistability and abrupt shifts in population size. We identify a broad range of dispersal rates in which populations show bistability and go either extinct or reach an equilibrium, depending on their initial population size. Crossing the limiting dispersal rates of this bistable regime, the system undergoes abrupt catastrophic shifts in population size, which would be overlooked under well-mixed assumptions. Our findings have broad biogeographic implications for predicting tipping points, hysteresis effects, and recovery from perturbations in mutualistic metacommunities.

## Introduction

Mutualistic interactions between species are ubiquitous in nature and can be critical for the stability of natural ecosystems as exemplified by cross-feeding microbes in the gut [1–3] and cooperative breakdown of sugar [4]. When one or more species of a community entirely rely on each other for survival, their populations must surpass a certain critical size to prevent extinction. This threshold property is often referred to as strong Allee effect at the community level (a *weak* Allee effect refers to scenarios where at low population size, the per capita growth rate decreases with decreasing population size, but never becomes negative). Models that incorporate a strong Allee effect are of great interest in ecology and are invoked frequently to explain tipping points accompanied by bistability and catastrophic shifts between survival and extinction in ecosystems [5–7]. As an instructive example, when microbes depend on each other in order to access vital resources, their population dynamics can exhibit a tipping point at which the community undergoes catastrophic shifts upon the variation of experimental parameters such as nutrient levels [4, 8–10].

**Fig 1:**
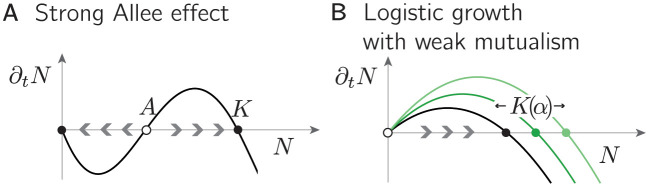
The (well-mixed) dynamics of a population with strong Allee effect vs logistic growth. **A.** If a population exhibits a strong Allee effect, its growth rate ∂_*t*_*N* is negative at low population sizes. The dynamics, therefore, heads toward extinction (*N* = 0) unless the initial population size exceeds a threshold, the Allee threshold *A*, upon which the population size rises to a limiting population size (*N* = *K*), referred to as carrying capacity. Full and open circles denote stable and unstable fixed points, respectively; arrows denote the flow of the dynamics. **B.** In contrast, a single-species population that undergoes regular logistic growth displays only one stable fixed point at the carrying capacity, while the extinct state is unstable. Additional mutualistic interactions, here tuned by a parameter *α*, can increase the effective carrying capacity *K*(*α*) without qualitatively changing the fixed point structure.

Although any community contains several populations, its dynamics can often be understood in terms of the one-dimensional dynamics of a single effective population (e.g. see [10]), illustrated in Fig 1A. Strong Allee effects induced by obligate mutualism between species therefore generate a very similar dynamics and threshold phenomena as intra-specific strong Allee effects, which can be observed in natural populations of all length scales, including zooplankton [11], plants [12], and polar bears [13].

However, strong Allee effects and the associated tipping points and bistability only occur in well-mixed communities if the mutual interactions are vital (i.e. obligate) for the survival of the community. In fact, well-mixed ecosystems with weakly mutualistic interactions (facultative mutualism) behave very similar to entirely non-mutualistic ecosystems, except that the carrying capacity of their logistic growth is enhanced due to the mutualistic interactions between different species (see Fig 1B).

It is known that the impact of strong Allee effects can be qualitatively different to that of logistic growth in spatially structured populations. For example, metapopulations consisting of patches with local Allee effects show pushed instead of pulled waves in range expansions [14–16], localized wave fronts [17], and pronounced patchiness [18]. However, it is currently unclear whether spatial structure can significantly alter ecosystem dynamics when mutualistic interactions are *weak*.

Here, we show that when demographic fluctuations are taken into account, even the weakest form of mutualism between species in a metacommunity can lead to a tipping point accompanied by bistability and catastrophic shifts between coexistence and extinction. Our combined analytical and numerical methods reveal a strong Allee effect in the metacommunity despite the absence of a strong Allee effect in the single patch dynamics. This *metacommunity-wide* strong Allee effect leads to range of intermediate dispersal rates where species can avoid extinction when the mean population size overcomes a threshold value. Informed by our intuition regarding purely mutualistic interactions, we show that close to the shift from extinction to finite population sizes, metacommunities with random interactions undergo selection for mutualistic interactions. We further apply our analyses to metacommunities in which interactions between species increase their dispersal instead of their growth rate and find a similar emergent tipping point. Our results give insights into the role of demographic stochasticity and dispersal in metacommunities and highlight the emergence of tipping points and catastrophic shifts even when absent under well-mixed conditions.

## Mathematical approach

In the following, we consider *S* species that live in a metacommunity of *P* coupled communities (patches), where *P* is assumed to be large. The dynamics of the population size *N*_*x*,*i*_ of species *i* ∈ {1, …*S*} on patch *x* ∈ {1, …, *P*} is modeled by the following set of generalized Lotka-Volterra equations:

$$\begin{array}{cc} \partial_{t}N_{x,i}\left(t\right) & =rN_{x,i}(1-\frac{N_{x,i}}{K}+\frac{\alpha }{K}\sum_{j,j\neq i}^{S}N_{x,j})\\ & +\lambda \left({\bar{N}}_{i}-N_{x,i}\right)+\sqrt{N_{x,i}}\;\eta_{x,i}\;. \end{array}$$ (1)

The first term in Eq (1) describes growth of a species’ population at a growth rate *r* > 0, which saturates at a carrying capacity *K* due to self-limiting interactions. The interaction parameter *α* > 0 denotes the strength of mutualistic interactions between species. Assuming a constant interaction strength *α* between all species allows an analytic mean-field description; the results of this analysis will yield important intuitions when we later allow variations in the species’ inter-species interactions. The second term in Eq (1) takes into account dispersal, where we assumed, as a simple spatial approach, that all patches are connected through dispersal with a species-independent dispersal rate λ and ${\bar{N}}_{i}$ denotes the abundance of species *i* averaged over all *P* patches. The last term in Eq (1) reflects demographic fluctuations due to random births and deaths of individuals within a population, where *η*_*x*,*i*_ denotes uncorrelated noise with zero mean and unit variance. The square-root dependence of demographic noise on the density ensures that the expected population size variance is proportional to the expected number of birth or death events occurring in a given population [19, 20] (setting the amplitude of noise to unity amounts to setting the unit of time to be about one generation time). Considering only the deterministic patch dynamics without migration, i.e. the the first line of Eq (1), all species display the same population dynamics. When we further assume that mutualistic interactions are weaker than self-limiting interactions, i.e. *α* < (*S* − 1)^−1^, (to ensure that population sizes do not diverge) all species have an unstable state at zero population size and a stable state with abundance N* given by

$$\begin{array}{c} N^{*}=K/\left[1-\alpha \left(S-1\right)\right]\;. \end{array}$$ (2)

The fixed point structure of each species on an isolated patch thus resembles regular logistic growth with a carrying capacity N* that increases with *α*, as illustrated in Fig 1B. Specifically, this means that population growth on an isolated patch does *not* exhibit a strong Allee effect nor bistability. In the following, we will solve Eq (1) numerically and employ mean-field analyses to study the effect of demographic fluctuations and dispersal and show how these can, nevertheless, generate bistability and an abrupt shift in the population size.

## Results

### Weak mutualism generates a tipping point in a metacommunity

**Fig 2:**
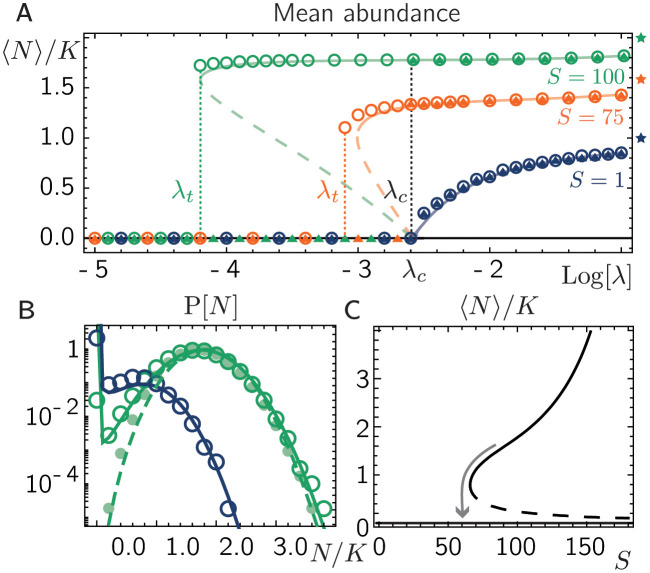
Weak mutualism generates a tipping point. **A.** Starting at small and large initial population sizes (triangles and circles, respectively), the mean deme population size 〈*N*〉 in our numerical solutions settles at a positive value or decays to zero. The long term steady state values are in very good agreement with our mean-field solution (lines). Solid and dashed lines denote stable and unstable manifolds, respectively. Colors denote different numbers *S* of species as indicated. The analytical result for λ_*c*_ is shown as black dotted vertical line and the *S*–dependent tipping point dispersal rates λ_*t*_ are indicated by vertical dotted lines of corresponding color. The deterministic steady states N*, given by Eq (2), are indicated by stars on the right next to the plot. **B.** Numerical and analytic solutions for the abundance distribution *P*[*N*] (circles and lines, respectively) for *S* = 100 with λ < λ_*c*_ (λ = 10^−4^, green open circles and solid line) and λ > λ_*c*_ (λ = 10^−1^, green full circles and dashed line) as well as for *S* = 1 with λ closely above λ_*c*_ (λ = 10^−2.4^, blue). **C.** Small changes in the species number, i.e. through perturbations, can lead to a collapse of the metacommunity (as indicated by the arrow), λ = 0.001. Parameters: *r* = 0.3, *K* = 10, *α* = 0.005, *P* = 500.

For a clearer presentation of our results, in the following we fix *r*, *K*, and *α* (with *α* ≪ (*S* − 1)^−1^), and vary the dispersal rate λ for different numbers of species *S*. First, we discuss our numerical solutions of Eq (1) assuming small average abundances 〈*N*〉 = (*SP*)^−1^ ∑_*x*,*i*_ *N*_*x*,*i*_ as initial condition (for details on the numerical solution, see S1 Text, Sec. S1). The impact of growth, demographic fluctuations and dispersal on single species has been extensively studied for short-range dispersal in the context of spreading processes and transport in random media within the theory of directed percolation [21, 22] and for global dispersal in metapopulations [23–26]. From these earlier studies we expect that for *S* = 1, increasing the dispersal rate leads to a continuous transition from a phase of zero population size (absorbing phase) to a phase of non-zero population sizes (active phase). Indeed, when numerically solving the dynamics Eq (1) with global dispersal and for only one species (*S* = 1), we find that for zero and small dispersal rates λ the species eventually goes extinct, i.e. the population sizes on all patches are zero (without return). In contrast, for λ above a critical threshold value λ_*c*_, the average population size after the final time step of our numerical solution is positive and increases continuously with λ (see triangles in Fig 2A) while being bounded from above by the deterministic carrying capacity N* given by Eq (2). Interestingly, when increasing the number of species at constant mutualistic interaction strength *α* > 0, the average abundance as a function of the dispersal rate λ undergoes a sudden jump at λ_*c*_ from zero to positive values. Discontinuous transitions are a telltale sign of subcritical bifurcations and bistability close to the transition [27]. To investigate the possibility of multiple stable solutions, we repeat our numerical solutions for larger initial average abundances, and, indeed, find bistability close to the threshold dispersal rate λ_*c*_ for larger *S* (see circles in Fig 2A).

To get deeper insights into the exact form of the bifurcation, we employ a mean-field approximation of Eq (1), that has been recently presented to approximate the stationary abundance distribution for metacommunities with competitive interactions [23]. This mean-field analysis allow us to consider metacommunities with an infinite number of patches and thereby circumvent possible finite size effects of our numerical analysis. In short, by expressing the interaction term through the species-averaged abundance on a patch defined as ${\hat{N}}_{x}=S^{-1}\sum_{i}N_{x,i}$ and treating the mean-fields ${\hat{N}}_{x}$ and ${\bar{N}}_{i}$ as deterministic mean-field parameters, we can map the dynamics Eq (1) to the solvable problem of a Brownian particle in a fixed potential. Due to the statistical equivalence of all populations for all species and patches, we can demand that ${\bar{N}}_{i}$ and ${\hat{N}}_{x}$ are both equal in equilibrium (in the limit of an infinite number of species and patches). This eventually allows us to derive an analytic expression for the abundance distribution as a function of the mean species abundance $\langle N\rangle \equiv {\bar{N}}_{i}={\hat{N}}_{x}$ and the control parameters *r*, *K*, *α*, *S*, and λ, which can be solved self-consistently (for a detailed derivation see [23] and S1 Text, Sec. S2).

In agreement with our numerical solution, our analytic mean-field approximation predicts bistability between the dispersal rate λ_*c*_ (for an analytic expression see [23] and S1 Text, Sec. S2.1) and a lower dispersal rate λ_*t*_. The rate λ_*t*_ marks a point where a small decrease of the dispersal rate causes an abrupt shift from positive population size to extinction, often referred to as tipping point [7] (see vertical dotted lines in Fig 2A). In addition, our analytic solution reveals an unstable branch marking the threshold mean abundance as a function of the dispersal rate the metacommunity must exceed to reach a non-zero mean abundance and avoid extinction (see dashed lines in Fig 2A). We also obtain the complete abundance distribution P[N] in analytical form, which has main contributions from extinct species (*N* = 0) for small λ and a maximum close to N* (see Fig 2B, for details see S1 Text, Sec. S2).

In summary, our numerical and analytical results strongly suggest that despite the lack of bistability in the deterministic and well-mixed population dynamics, the metacommunity displays a tipping point accompanied by a regime of bistability. The identified bifurcation predicts a discontinuous transition from extinct to colonized as the migration rate surpasses λ_*c*_, and from colonized to extinct when the dispersal rate is lowered towards the tipping point λ_*t*_. The population goes through a hysteresis loop if the migration parameter cycles between a value below λ_*t*_ and above λ_*c*_.

The emergence of bistability for low migration rates can be rationalized in two steps. First, note that when the species’ patch population sizes are sufficiently small (〈*N*〉≪1), the probability for two or more species to encounter each other on the same patch becomes small. This decrease in encounters is significant because interactions between species, which are nonlinear, depend on these encounters. Thus, in situations where population density is low, these species-to-species interactions can be reasonably ignored. Consequently, the extinct state is stable if the species’ dispersal rates are below the critical threshold for individual species survival (λ_*c*_), irrespective of the number of species involved. The question of bistability then boils down to the question of whether, for λ < λ_*c*_, there is another stable state—besides the extinct state. Such a stable state is possible if the species interactions allow the population to survive. Intuitively, this is possible at high enough densities so that the mutualistic interactions, as reflected by the positive contribution to the growth term in Eq (1), compensate for the noise-driven decay. In our phase diagram this occurs if the densities lie above the dashed line in Fig 2A (see S1 Text, Sec. S2.2, and S2 Fig).

We find that the range of bistability increases the more species interact through mutualism (see Fig 2C). As a consequence, this suggests that perturbations that decrease the number of species, even if only by a few species, may shift the metacommunity into a regime where eventually all species go extinct (see arrow in Fig 2C).

While we find that demographic fluctuations combined with spatial structure can result in bistability, previous studies suggest that demographic fluctuations can also produce the opposite effect on population transitions: a single species metapopulation, which displays bistability under deterministic and well-mixed conditions (imposed through an explicit strong Allee effect on every patch), may experience a gradual transition when dispersal and stochasticity are taken into account (see [28–30]). In S1 Text, Sec. S3, we investigate this prior observation by means of our mean-field approach applied to a metapopulation model of a single species that shows bistabilty on every patch. Consistently with the above mentioned studies, we find that when increasing the dispersal rate, the system undergoes a continuous transition from extinction to positive population sizes. While these results show that stochasticity can generally smoothen discontinuous transitions, our study on mutualistic metacommunities highlights the interesting and somewhat opposite role of stochasticity when it occurs in combination with species interactions: here, it can lead to discontinuous transitions even when they are absent in the deterministic dynamics.

### Spatial structure selects for metacommunities with an excess of mutualistic interactions

The dramatic change from a smooth to a discontinuous transition close to the threshold dispersal rate λ_*c*_ suggests several implications for more general species interactions. Since in contrast to species with competitive interactions, species with mutualistic interactions are able to reach large finite abundances already well below λ_*c*_, we hypothesize that in a metacommunity with random interactions, mutualism may play an important role in community assembly, at least close to λ_*c*_. To test this idea, we generalize the metacommunity dynamics Eq (1) and assume random interactions *α*_*i*,*j*_ between species *i* and *j*. The generalized metacommunity dynamics reads

$$\begin{array}{cc} \partial_{t}N_{x,i}\left(t\right) & =r_{i}N_{x,i}(1-\frac{N_{x,i}}{K}+\sum_{j,j\neq i}^{S}\frac{\alpha_{i,j}}{K}N_{x,j})\\ & +\lambda \left({\bar{N}}_{i}-N_{x,i}\right)+\sqrt{N_{x,i}}\;\eta \;, \end{array}$$ (3)

where we again assumed global dispersal between patches. For a clearer distinction between mutualistic and competitive interactions between species, we choose interactions to be symmetric, i.e. *α*_*j*,*i*_ = *α*_*i*,*j*_. Motivated by earlier work on well-mixed communities [31–36], we draw *α*_*i*,*j*_ from a Gaussian distribution with mean $\hat{\alpha }$ and standard deviation $\sigma /\sqrt{S}$. Hence, it is possible to choose $\hat{\alpha }$ and *σ* in a way that interactions between some species *i* and *j* are mutualistic (i.e. *α*_*i*,*j*_ > 0) while interactions are on average competitive ($\hat{\alpha }<0$). A detailed analytical understanding of the spatially structured community assembly with random interactions is beyond the scope of our mean-field analysis, but can be obtained using the replica method [37]. In the following we use numerical solutions of Eq (3) to show that, below the critical threshold dispersal rate λ_*c*_, communities survive that are enriched in mutualistic interactions, even though the interactions among the initial species pool are on average competitive.

**Fig 3:**
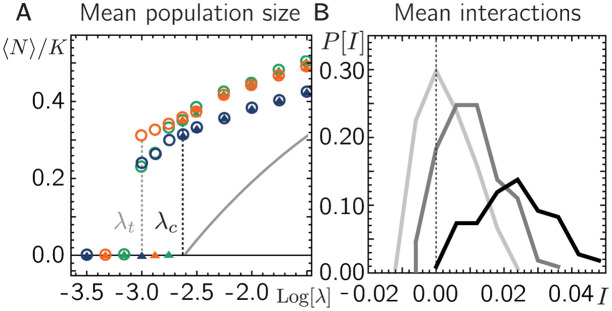
Tipping point for metacommunities with random interactions. **A.** Numerical solutions of Eq (3) for initially low (triangles) and high (circles) mean population sizes for three different sets of random interactions (denoted by three different colors) suggest bistability and hysteresis between the tipping point (gray dotted vertical line) and close to λ_*c*_ (black dotted vertical line). Gray solid line shows mean-field solution for identical interactions ($\alpha_{i,j}=\hat{\alpha }$) **B.** Distributions of mean interactions with other species for all three sets of random interactions shown in A for λ = 10^−2.8^, λ = 10^−2.2^, λ = 10^−1.5^ from dark to light, respectively (λ_*c*_ ≈ 10^−2.6^). Remaining Parameters: $\sigma =0.5,r=0.3,S=100,\;P=500,\;\hat{\alpha }=-0.01$ (on average competitive interactions).

First, we solve the dynamics Eq (3) numerically for fixed *r*, *K*, *S*, *σ*, negative mean interaction $\hat{\alpha }$ (i.e. an on average competitive interaction between species), and varying λ. Here, we will focus on relatively small interaction differences, specifically σ≲1, where previous studies suggest that, under well-mixed conditions, a community approaches a unique equilibrium state [33–35]. For larger *σ*, we expect the system to be multistable due to interspecies interactions alone (see also S1 Text, Sec. S4)–an effect that may obscure the emergence of bistability due to demographic noise and dispersal, which is our main focus here. Similar to our results for purely mutualistic interactions (Fig 2A), we find positive population sizes already for dispersal rates below λ_*c*_ (see Fig 3A). Furthermore, we observe bistability, i.e. for dispersal rates below λ_*c*_ the metacommunity approaches positive mean population sizes when the initial population size is sufficiently large while it goes extinct otherwise. We comment that in contrast to our model of equal mutualistic interactions, Eq (1), where all species either go extinct or survive together, differences in the species’ interactions generally lead to extinctions of some species in our numerical solution while the rest of the species persist. In the regime where species survive, the final mean population size (see different colors in Fig 3A) and the number of species that go extinct (see S1 Text, Sec. S4) depend on the chosen set of random interactions and generally increase with the dispersal rate. While we focused on purely symmetric interactions, based on [37] we expect these results to hold even in the presence of moderate asymmetric contributions in the interaction coefficients.

In order to investigate the role of mutualistic interactions especially in the regime of dispersal rates around λ_*c*_, we calculate the average interaction *I*_*i*_ ≔ 〈(*α*_*i*,*j*_/*K*)*N*_*x*,*j*_〉_*i*_, where the average is taken over all patches *x* and co-surviving species *j* at the last time point of our numerical solution (see Fig 3B). In line with our intuition from purely mutualistic interactions, we find that for dispersal rates below λ_*c*_, all species that survive form on average mutualistic interactions with their fellow surviving species. (for λ < λ_*c*_, the distribution of *I*, P[I], in Fig 3B has only contributions from positive *I*).

When we increase the dispersal rate beyond λ_*c*_, more species survive and, in particular, also species with on average competitive interactions coexist. We were also interested how the communities of remaining survivors of our numerical solution compare to our mean-field model with species-independent interaction coefficient. To this end, we calculate the mean-field solution with the number of species *S* and the species-independent interaction coefficient *α* being equal to the number of surviving species, *S*_surv_ and their average interaction coefficient, ${\hat{\alpha }}_{\text{surv}}$, respectively, that we obtained from our numeric simulation of the metacommunity with random interactions and different dispersal rates. Similar to Fig 2, we can now plot the steady states of the metacommunity based on the mean-field solution with the parameters *S* = *S*_surv_ and $\alpha ={\hat{\alpha }}_{\text{surv}}$ as a function of the dispersal rate λ. As shown in S1 Text, Sec. S4, and S5 Fig, the resulting bifurcation again shows a tipping point at some $\lambda_{t}^{*}$, marking the onset of a stable state with positive population size. Interestingly, the dispersal rate $\lambda_{t}^{*}$ of this tipping point is close to the dispersal rate of the underlying numerical solution. Heuristically, this suggests that in the assembly process of a community with random, predominantly competitive interactions, species will die out and adapt their population sizes until the remaining community reaches a state which is just viable for a given dispersal rate (i.e. so that the tipping point dispersal rate $\lambda_{t}^{*}$ is close to the dispersal rate λ). As a result, random metacommunities with predominantly competitive interactions self-organize to a state very close to a tipping point, where survival of the metacommunity may be very sensitive towards perturbations in its parameters, e.g. the dispersal rate.

### Tipping point through density-dependent dispersal

So far, we have incorporated mutualism through direct interactions between species, such that interactions effectively increase the species growth [see Eq (1) and Eq (3)]. In the following, we investigate interactions between species through their dispersal, i.e. interactions that increase the species’ dispersal rates.

**Fig 4:**
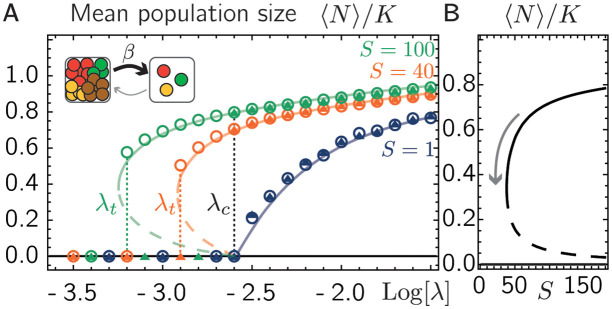
Density-dependent dispersal generates tipping point. **A.** The mean population size in our numerical solutions can reach different values when starting at small and large initial population sizes (triangles and circles, respectively) in agreement with our mean-field solution (solid and dashed lines denote stable and unstable manifolds, respectively). The black dotted vertical line marks λ_*c*_, the colored dotted vertical lines lines indicate λ_*t*_ based on numerical solutions. The inset illustrates density-dependent dispersal, where emigration increases with the abundance of individuals from other species on a patch. **B.** Mean-field solution of the mean abundance predicts a catastrophic shift as function of the species number, λ = 0.001. Remaining parameters: *r* = 0.3, *K* = 10, *β* = 0.02, *P* = 500.

There is indeed evidence in many species [38–42] that emigration rates from crowded areas tend to be elevated to avoid competition for resources. Since this effectively results in a dispersal rate that increases with the abundance of other populations, we refer to this scenario as interactions through density-dependent dispersal in the following. When assuming that, as a first approximation, emigration from a patch increases linearly with the number of individuals of other species already present, the dispersal term in Eq (1) can be written as

$$\begin{array}{c} \frac{\lambda }{P}\;[\;\sum_{y}^{P}\;(\;1+\beta \sum_{j\neq i}^{S}N_{y,j}\;)\;N_{y,i}-(\;1+\beta \sum_{j\neq i}^{S}N_{x,j}\;)\;N_{x,i}]\;, \end{array}$$ (4)

where we assumed a constant baseline dispersal rate λ between every patch and a linear increase of dispersal with population size with a factor *β*. Next, we solve Eq (1) for fixed *r*, *K*, and *S* > 1 with *α* = 0, i.e. without direct mutualistic interactions, and the dispersal term Eq (4) with *β* > 0 numerically and discuss the respective mean-field approximation (for details on the mean-field description, see S1 Text, Sec. S2.3). Similarly to direct mutualistic interactions between species, we find that when varying the baseline dispersal rate λ, the average abundance of the metacommunity undergoes a sudden shift at λ_*c*_ (see Fig 4A). As for direct mutualistic interactions, the regime of dispersal rates λ that shows bistability grows for increasing *S* see Fig 4B). We thus conclude that interactions that increase species’ dispersal with the population sizes on a patch result in a similar phenomenology as interactions that increase the growth of the species’ populations on a patch, including catastrophic shifts and a metacommunity-wide strong Allee effect.

Intuitively, bistability can emerge from density-dependent dispersal since for λ < λ_*c*_, sufficiently large population sizes can elevate the realized dispersal rate above the threshold value λ_*c*_ (this is in agreement with our observations, compare S1 Text, Sec. S2.3, and S2 Fig]).

We comment that the observed mutualism and tipping point that result from density-dependent dispersal persist even in the presence of additional direct competition between species, albeit the regime of bistability shrinks with increasing strength of direct competition (see S1 Text, Sec. S2.3, and S3 Fig).

## Discussion

While it is well-established that strong (obligate) mutualism, which leads to strong local Allee effects, can create complex behaviors in spatially structured systems, our findings reveal that even the weakest form of mutualism among species on a patch can have profound impacts. These effects would be overlooked in a conventional, well-mixed deterministic framework. Specifically, weak mutualism can greatly influence the stability of a metacommunity, leading to a *metacommunity-wide* strong Allee effect, characterized by tipping points and hysteresis. As a result, abrupt transitions between species coexistence and complete metacommunity collapse can be induced by slight variations in factors like dispersal rate, the number of interacting species, or the strength of mutualistic interactions, for instance, due to environmental changes.

Previous research has demonstrated that mutualistic interactions enhancing population growth across different patches in a metapopulation [29] and memory effects in a metacommunity [43] can lead to Allee thresholds along with critical tipping points. Our study adds to this body of work by illustrating that even a basic form of mild mutualistic interactions can induce thresholds at the metacommunity level. It is important to note that the pronounced Allee effect observed in our metacommunity is not a predefined part of our model, unlike previous metapopulation models that explicitly include Allee thresholds [44–46], but rather a property that emerges from our metacommunity model.

The appearance of bistability in our stochastic model contrasts with the typical smoothing effect of demographic noise ([28, 30] and S1 Text, Sec. S3) and illuminates how demographic fluctuations can act as a catalyst for abrupt transitions when they interact with interspecies relationships.

In communities with random interactions we found that limiting dispersal tends to favor the mutualistically interacting part of the community. Previous studies suggest, that under well-mixed conditions community assembly selects against competitive and enriches mutual (beneficial) interactions [47]. In our metacommunity model, survival poses a much stronger requirement: for dispersal rates below λ_*c*_, only communities with on average mutualistic interactions can survive if they are large enough.

Our results for random interactions suggest various interesting directions for future studies that depart from our assumption of small symmetric differences in the interaction coefficients. These include metacommunities with larger differences in the interaction coefficients, which have been suggested to yield multistability even in a well-mixed scenario [34], as well as metacommunities with asymmetric interaction statistics, which can lead to fluctuating steady state and even chaos in a deterministic metacommunity [48, 49]. The relevance of our main results for more complex models advocates further study of simplified models that can help to understand isolated features of complex ecological systems while offering a more feasible mathematical approach and interpretation.

We found qualitatively similar effects in a system where interactions between species act on the dispersal rate of species in such a way that emigration from a patch increases with the population size of other species present. From a technical point of view, the similarity of both types of interaction comes from the fact that an increase in both growth rate and dispersal rate should have a positive effect on a species persistence. In a more ecological context, dispersal that increases with the densities of other species on a patch can arise from competition between species and can thus be interpreted as effectively competitive interaction. Following this interpretation, our results suggest that competition that causes avoidance of species on a patch can eventually lead to a mutualistic effect between species on the metacommunity level, suggesting a more general view of mutualism in metacommunities.

## Methods

### Numerical solution of the metacommunity dynamics

To numerically solve the metacommunity dynamics described by Eqs (1), (3) and (4), we employed a numerical update scheme where for every time step Δ*t* we first calculate the deterministic contributions (i.e. growth, competition and dispersal of every species on every patch) based on a Euler forward method. After that, demographic fluctuations are implemented by drawing the updated abundances from a Poisson distribution, which ensures the right statistics for the stochastic contributions. All calculations were performed in Python [50] and the results were evaluated using Mathematica [51] (the Python code developed for this study is available at https://github.com/Hallatscheklab/Self-Consistent-Metapopulations). For a more detailed description of the numerical methods see S1 Text, Sec. S1.

### Mean-field approach

Complementary to the numerical solution, we employed a mean-field theory where the species-averaged and patch-averaged abundances are approximated by their mean-field values $\hat{N}$ and $\bar{N}$, respectively. As detailed in the main text and S1 Text, Sec. S2, this mean-field approximation allows us to derive the equilibrium species abundance distribution of (1), P, as a function of the mean-fields $\hat{N}$ and $\bar{N}$. Finally, we numerically calculate $\hat{N}$ and $\bar{N}$ by demanding self-consistency, i.e. $\hat{N}=\bar{N}={\langle N\rangle }_{P}$, where ${\langle N\rangle }_{P}$ denotes the mean abundance based on the distribution P (all calculations were performed using Mathematica [51]). Applying this mean-field analysis to Eq (1) (see S1 Text, Sec. S2.1), to metacommunities with a density-dependent dispersal given by Eq (4) (see S1 Text, Sec. S2.3), and the observed parameters from numerical solutions with random interactions (see S1 Text, Sec. S4), we can derive the bifurcations, *i.e*. the mean population size as a function of the dispersal rate λ, discussed in Figs 2 and 4, and in the context of random interactions, respectively.

## Supporting information

S1 Text Supporting information. Detailed explanations of the numerical approach and analytical mean-field analyses including explanations of the emergent bistability and extended analyses of metacommunities with random interactions as well as metacommunities with density-dependent dispersal with additional direct competitive interactions.
(PDF)

S1 Fig Self-consistency condition in the mean-field approximation. (EPS)

S2 Fig Species interactions can raise the species growth and dispersal parameters and thereby enable survival. (EPS)

S3 Fig Moderate direct competition does not alter the emergence of a tipping point in a metacommunity with density-dependent dispersal. (EPS)

S4 Fig Smooth and discontinuous transitions in metapopulations with explicit strong Allee effect. (EPS)

S5 Fig Bistability and self-organized tipping points in random metacommunities. (EPS)
