# Supplementary material for: Tipping points emerge from weak mutualism in metacommunities
Source: PLoS Comput Biol. 2024 Mar 5;20(3):e1011899. doi: 10.1371/journal.pcbi.1011899 (PMC10942259; doi:10.1371/journal.pcbi.1011899)
Supplement: S1 Text — Detailed explanations of the numerical approach and analytical mean-field analyses including explanations of the emergent bistability and extended analyses of metacommunities with random interactions as well as metacommunities with density-dependent dispersal with additional direct competitive interactions. (PDF) [file pcbi.1011899.s001.pdf]

Tipping points emerge from weak mutualism in metacommunities – Supporting Information

Jonas Denk[1,2], Oskar Hallatschek[1,2,3,*]

1 Department of Physics, University of California, Berkeley, CA 94720, USA
2 Department of Integrative Biology, University of California, Berkeley, CA 94720, USA
3 Peter Debye Institute for Soft Matter Physics, Leipzig University, 04103 Leipzig, Germany

* ohallats@berkeley.edu

S1 Numerical solution of the metacommunity dynamics

As detailed in the main text, the metacommunity is assumed to follow the dynamics

$$\partial_t N_{x,i}(t) = rN_{x,i}\left(1-\frac{\alpha}{K}\sum_{j,j\neq i}^{S} N_{x,j}-\frac{N_{x,i}}{K}\right)+\sum_{y}^{P}\lambda_{y,x}(N_{y,i}-N_{x,i})+\sqrt{N_{x,i}}\,\eta\,, \quad \text{(S1)}$$

where $N_{x,i}$ denotes the abundance of species $i \in \{1,\dots S\}$ on the patch $x \in \{1,\dots,P\}$. The first term denotes population growth and interactions with other species, the second term denotes dispersal between patches, and The third term in general accounts for demographic fluctuations where η is uncorrelated noise with zero mean and variance ω^2. By rescaling the growth rate and the dispersal rate with the rate ω, we measure time in units ω^{-1} and can set $\omega = 1$ in the following (as also used in the main text). Unless noted otherwise, for Fig 2 and 3 in the main text we fixed the growth rate ($r = 0.3$), the competition strength ($\alpha = 0.005$), the carrying capacity ($K = 10$), and solved the dynamics for global dispersal for various dispersal rates λ and different numbers S of initially coexisting species based on the following Euler forward scheme. All calculations were performed in `Python` [1] and the results were evaluated using `Mathematica` [2]. For each time step Δt, we first calculate the update of the population size for each species on each patch given by the growth and dispersal dynamics [first two terms in Eq. (S1), respectively]. The contributions from growth and dispersal are calculated and updated separately in order to avoid unphysical scenarios, e.g. that an unoccupied patch acts as a source of dispersal. After updating the deterministic abundance of each species on each patch, demographic fluctuations [last term in Eq. (S1)] are added by sampling from a Poisson distribution with the mean being the deterministic abundance. Interpreting Eq. (S1) in the Itô sense [3], the Euler forward update for the demographic fluctuations are then incorporated by adding $\sqrt{\Delta t}(\texttt{Poisson}[N_{x,i}] - N_{x,i})$ to the deterministic abundances, where $\texttt{Poisson}[N_{x,i}]$ is a sample from a Poisson distribution with mean $N_{x,i}$. The implementation of demographic fluctuations through a Poisson process guarantees that their variance is given by $N_{x,i}$ [3], consistent with Eq. (S1). As initial condition we either choose $N_{x,i} = 15$ (large initial population sizes) or $N_{x,i} = 1$ (small initial population sizes) for all patches $x \in \{1,\dots P\}$ and species $i \in \{1,\dots S\}$ with additional small random fluctuations (fluctuations are drawn from a uniform distribution over the interval $[-5\%, 5\%]$ of the base initial value). For the numerical

solution of the more general metacommunity given by Eq. 3 in the main text in the main text we employ an analogous Euler forward scheme as above where the interaction strengths $\alpha_{i,j}$ are drawn from normal distributions centered around μ, with standard deviation σ/S. For our numerical solutions, the time steps Δt are adapted to values between 0.02 and 1 and the last time step is chosen to be 20000, measured in units ω^{-1}. The Python code developed for this study is mostly equivalent to the code of our previous study on metacommunities with competitive interactions available at `https://github.com/Hallatscheklab/Self-Consistent-Metapopulations`, where for mutualistic interactions, the sign of the interaction parameter α is changed.

S2 Self-consistent mean-field approach

In the following we will discuss our analytical mean-field approach to species-rich metacommunities with global dispersal that allows us to calculate static quantities such as the critical dispersal rate λ_c, the mean population size of species, and the abundance distribution. In contrast to our numeric solutions, our mean-field analysis enables us to consider a metacommunity with an infinite number of patches circumventing finite size effects (for instance, in our numeric solutions extinction is always expected if one waits long enough, where the time of extinction due to finite size is expected to increase exponentially with the number of patches.).

For convenience, we describe our analysis based on the dynamics for the rescaled abundances $f_{x,i} = N_{x,i}/K$. and introduce the mean fields $\bar{f}_i := P^{-1} \sum_x^P f_{x,i}$ and $\hat{f}_x := S^{-1} \sum_i^S f_{x,i}$, where $\bar{f}_i$ and $\hat{f}_x$ denote the averages of the rescaled abundance $f_{x,i}$ taken over patches and species, respectively. In the following we assume that the number of patches P and the number of coexisting species on each patch are large, and that all species are statistically identical [with identical growth rates, carrying capacities, interactions and dispersal rates, as in Eq. (S1)]. Under this assumption, we can expect that in the sums of $f_{x,i}$ over different patches and species in Eq. (S1) fluctuations between patches and species, respectively, cancel out approximately. We hence estimate the sums through the mean field expressions $\bar{f}_i$ and $\hat{f}_x$ and treat these mean fields as deterministic parameters. In the following, we will first present the mean-field description for the case of direct mutualistic interaction as in Eq. (S1). Later, we discuss an analogous solution for density-dependent dispersal with a dispersal term introduced in Eq. 4 in the main text.

S2.1 Mean-field approach for mutualistic interactions

Expressing Eq. (S1) through the mean fields $\bar{f}_i$ and $\hat{f}_x$, the dynamics for every species on every patch can be written as

$$\partial_t f(t) = f \frac{\partial \mathbf{F}}{\partial f} + \sqrt{f/K}\, \eta(t) \tag{S2}$$

$$\text{with: } \mathbf{F} = rf \left[1 - \frac{f}{2} + \alpha(S-1)\hat{f}\right] + \lambda \left[\bar{f}\log(f) - f\right] \,, \tag{S3}$$

where we omitted the species and patch index since all species and patches are assumed statistically identical. We comment that while the sums over $f_{x,i}$ over species or patches can be approximated by the mean fields $\bar{f}$ and $\hat{f}$, simply replacing f by the mean fields everywhere in Eq. (S2) would be wrong since we would thereby effectively ignore demographic fluctuations. The representation Eq. (S2) admits an analytical equilibrium distribution in terms of a Gibbs measure [4–7]. To see this, it is convenient to introduce

the variable $g := \sqrt{f}$. Using Itô's lemma, we can rewrite Eq. (S2) in terms of g as

$$\partial_t g = -\frac{\partial \mathbf{U}}{\partial g} + \frac{1}{2\sqrt{K}}\eta(t) \tag{S4}$$

$$\text{with: } \mathbf{U} = -\frac{1}{4}\mathbf{F} + \frac{1}{8K}\log(g)\,. \tag{S5}$$

This dynamics for g can be reinterpreted as the overdamped dynamics of a particle in a potential $\mathbf{U}$ with diffusion constant $1/(4K)$. Inspecting the potential $\mathbf{U}$, we see that the terms involving the logarithm of g lead to a divergence of $\mathbf{U}$ at $g = 0$, which is positive or negative for $2K\lambda\bar{f} - 1$ positive or negative, respectively. The remaining terms lead to a minimum at some positive g, which still depends on $\hat{f}$. As we will see below, these two contributions, the divergence at zero g and the minimum of $\mathbf{U}$ at positive g form the main contributions for the abundance equilibrium distribution. The equilibrium distribution $\mathcal{P}[g]$ for g is then given through the Gibbs measure

$$\mathcal{P}[g] \sim \mathrm{e}^{-8K\mathbf{U}} = \frac{1}{g}\mathrm{e}^{2K\mathbf{F}}\,, \tag{S6}$$

which is equivalent to a Boltzmann distribution with an "energy" given by $\mathbf{F}$.

In terms of the rescaled abundance f, we have $\mathcal{P}[f] \propto \frac{1}{f}\mathrm{e}^{2K\mathbf{F}}$. Equivalently, we can write

$$\mathcal{P}[f, \bar{f}, \hat{f}] = \frac{1}{Z}\frac{1}{f^{1-2K\lambda\bar{f}}}\mathrm{e}^{2Krf\left[1+\alpha(S-1)\hat{f}-\frac{f}{2}\right]-2K\lambda f}\,, \tag{S7}$$

where we omitted the dependence of $\mathcal{P}$ on r, K, α, and λ, and Z denotes the normalization constant such that $1 = \int_0^\infty P[f]\,\mathrm{d}f$[1] In terms of the abundance $N = Kf$, the distribution can be written as

$$\mathcal{P}[N, \bar{N}, \hat{N}] = \frac{1}{Z}\frac{1}{N^{1-2\lambda\bar{N}}}\mathrm{e}^{2rN\left(1+\alpha(S-1)\frac{\hat{N}}{K}-\frac{N}{2K}\right)-2\lambda N}, \tag{S8}$$

with respective normalization constant Z so that $1 = \int_0^\infty P[N]\,\mathrm{d}N$. As a consequence of the divergence and minimum of $\mathbf{U}$ discussed above, the main contributions of the abundance distribution $\mathcal{P}[N, \bar{N}\hat{N}]$ come from its divergence at $N = 0$ (when $1 - 2\lambda\bar{N}$ is positive), and some positive N, resulting from the maximum of the polynomial of second order in the exponent of Eq. (S8). Note, that the abundance distribution Eq. (S7) still depends on the parameters $\bar{f}$ and $\hat{f}$. While we treated the mean fields $\bar{f}$ and $\hat{f}$ as deterministic parameters, in order for our analysis to be self-consistent they have to be equal and also equal the actual statistical mean of f, which can be calculated from the distribution Eq. (S7). Introducing a Lagrange multiplier $+\epsilon f/2K$ into the function F we can take the derivative of $\log(Z)$ w.r.t to ϵ, take the limit $\epsilon \to 0$, and thereby obtain the mean abundance $\langle f\rangle_{\mathcal{P}[\bar{f},\hat{f},r,K,\alpha,\lambda]}$[2]. Self-consistency then requires:

$$\bar{f} = \hat{f} = \langle f\rangle_{\mathcal{P}} \tag{S9}$$

Fig S1A shows the calculated mean $\langle f\rangle_{\mathcal{P}}$ as a function of $\bar{f}$ (where for specificity $r = 0.3$, $K = 10$, $\alpha = 0.1$, and $\hat{f} = \bar{f}$ due to self-consistency). All calculations were performed using `Mathematica` [2]. Varying the dispersal rate λ we find that for small λ

[1] We comment, that for $\bar{f} = 0$ the probability distribution cannot be normalized due to the divergence of order $1/f$ at $f = 0$.

[2] To see this, first note that the derivative of $\log(Z)$ results in $(1/Z)\partial_\epsilon Z$. Taking the partial derivative w.r.t ϵ inside the integral over all f in Z, one obtains an additional factor of f in the integral. Taking the limit $\epsilon \to 0$, the whole expression amounts to the integral over the normalized abundance distribution times f and thus is equal to the mean of the rescaled abundance f.

the only solution to the self-consistency condition, Eq. (S9), is given by $\bar{f} = 0$. For $S = 1$, increasing λ above a critical value λ_c, the solution $\bar{f} = 0$ is no longer stable; however, there appears a second solution with non-zero $\bar{f}$, which is linearly stable and increases with λ [see Fig S1A]. Thus, λ_c marks a bifurcation from zero to non-zero mean abundances. For sufficiently large mutualistic interactions (e.g. S is sufficiently large for constant α), the self-consistency condition $\langle f \rangle = \bar{f}$ suggest an abrupt jump from zero to finite values close to the tipping point [see Fig S1B].

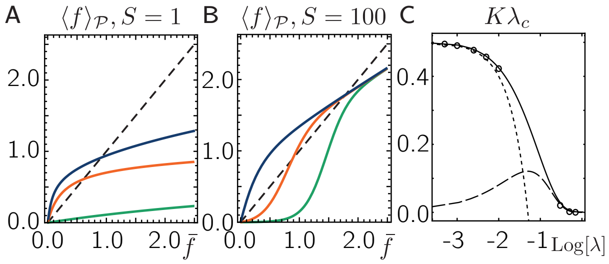

Fig S1. Self-consistency condition in the mean-field approximation. A. Above a critical dispersal rate, λ_c, the self-consistency condition $\langle f \rangle \equiv \bar{f}$ (dashed line) has a solution with non-zero mean abundance $\langle f \rangle$ marking an onset of finite population sizes. Shown are $\langle f \rangle$ for dispersal rates smaller (green, $\lambda = 10^{-3}$), close above (orange, $\lambda = 10^{-2}$) and farther above (blue, $\lambda = 10^{-1}$) the critical dispersal rate λ_c. **B.** For sufficiently large mutualistic interactions (here, $S = 100$, $\alpha = 0.005$), there is a discontinuous transition at the tipping point from a solution with zero to a solution with finite mean abundances. Shown are $\langle f \rangle$ for dispersal rates smaller (green, $\lambda = 10^{-5}$), close above (orange, $\lambda = 10^{-3.5}$) and farther above (blue, $\lambda = 10^{-2}$) the tipping point. Remaining parameters: $r = 0.3$, $K = 10$. **C.** The self-consistent mean-field solutions for the critical dispersal rate $\lambda_c(r)$ (solid line) are in very good agreement with our numerical solutions (open circles). The dashed and dotted lines denote the limiting behaviors for $K\lambda/(Kr) \ll 1$, Eq. (S11), and $Kr/(K\lambda) \ll 1$, Eq. (S12), respectively. Remaining parameters: $K = 10$, $S = 1$, and for the numerical solutions: $P = 2000$.

Critical dispersal rate. To obtain an analytical expression for the critical dispersal rate we expand the calculated mean $\langle f \rangle_{\mathcal{P}[\bar{f}]}$ to first order in $\bar{f}$. This yields the condition for the onset of finite mean abundance:

$$\mathrm{e}^{\frac{(Kr-K\lambda)^2}{Kr}} \frac{K\lambda}{\sqrt{Kr}} \sqrt{\pi} \left(1 + \mathrm{Erf}\left[\frac{Kr - K\lambda}{\sqrt{Kr}}\right]\right) \stackrel{!}{=} 1\,, \tag{S10}$$

where Erf[·] denotes the Error-function (incomplete Gaussian integral). Note that the onset of finite mean abundances, Eq. (S10), does not depend on the interaction strength α nor the number of interacting species S. This is consistent with the expectation that at the onset of finite mean abundances, interactions between species on a patch should be negligible. For the limiting case $K\lambda \ll Kr$, we expand the condition Eq. (S10) up to first order in $K\lambda/(Kr)$ and solved for λ, which yields the critical dispersal rate λ_c:

$$K\lambda_c(r, K) \approx \mathrm{e}^{-Kr} \sqrt{\frac{Kr}{\pi}} \left(1 + \mathrm{Erf}[\sqrt{Kr}]\right)^{-1}\,. \tag{S11}$$

When furthermore $K\lambda \ll 1$, the growth rate must be correspondingly large so that we can set $\mathrm{Erf}[\sqrt{Kr}] \approx 1$. This yields $\lambda_c(r, K) \approx \mathrm{e}^{-Kr}\sqrt{\frac{r}{4K\pi}}$. The observation of a finite dispersal threshold for global dispersal is consistent with previous studies of metapopulations with implicit spatial extension [8–10], which used master equations to

model species birth, death and global dispersal between patches through a shared reservoir. Note that the critical dispersal rate decreases exponentially with increasing K. For large K, stochastic fluctuations become less relevant in the dynamics of all species; this is best seen in the dynamics of the rescaled densities, Eq. (S2), where for large K, the coefficient in front of the stochastic term goes to zero. As a result, the dispersal rate to counteract stochastic extinctions, λ_c, decreases. The regimes of stochastic extinctions and bistability will thus persist, albeit at lower dispersal rates. In the limit of infinite K, stochastic effects become negligible and the system behaves deterministically. In this case, all abundances $N_{x,i}$ for all species and patches will approach the steady state solution given by Eq. 2 of the main text.

In the limiting case $K\lambda \gg Kr$ we can expand the condition Eq. (S10) to leading order of large $K\lambda/(Kr)$, and solve for λ. This yields the approximation:

$$\lambda_c(r, K) \approx \frac{1}{2K} - r\,, \tag{S12}$$

Hence, we find that for infinitesimally small finite growth rates r, the critical dispersal rate approaches $\lambda_c(r, K) = 1/(2K)$. Both limiting behaviors of the critical dispersal rate λ_c, at $K\lambda/(Kr) \ll 1$ and $Kr/(K\lambda) \ll 1$, are in very good agreement with respective numerical solutions of the Langevin equation 1 of the main text [see Fig S1,C]. While the critical dispersal rate λ_c can be derived by solving the condition that the first derivative of the statistical mean w.r.t. the mean $\bar{f}$ is zero, deriving an expression for λ_t is much less feasible. Especially, in contrast to the derivation of λ_c, there is no simple condition on the statistical mean or its derivatives that would guarantee that the identity line and the line of the statistical mean cross for positive $\bar{f}$.

Equilibrium abundance distribution. Beyond the onset of finite mean abundances ($\lambda > \lambda_c$), we can solve for the mean abundance $\bar{f}$ that satisfies the self-consistency condition Eq. (S9) numerically [see Fig S1A,B]. Eventually, substituting this numerical solution for $\bar{f}$ into Eq. (S7) yields the equilibrium abundance distribution $\mathcal{P}$ as a function of r, K, α, and the dispersal rate λ. The derived abundance distribution is governed by different contributions, depending on the choice of parameters: When the dispersal rate is small ($\lambda K\bar{f} \ll 1$) and for $f \ll 1$ (i.e. abundances $N \ll K$), the distribution of the rescaled abundance f follows the scaling

$$\mathcal{P}[f] \propto x^f/f \text{ with } x = \mathrm{e}^{-2K\left[r(1+\alpha(S-1)\bar{f})-\lambda\right]}\,. \tag{S13}$$

The form of the abundance distributions $\mathcal{P}[f] \propto x^f/f$ (with x being a system-dependent parameter) is well-known in ecology literature as Fisher log series [11], which denotes one of the most widely used abundance distributions in ecology and has been recovered in a variety of ecological systems (see [12, 13] for reviews). For larger rescaled abundances ($f \sim 1$), the exponential term in Eq. (S7) displays a local maximum of the abundance distribution characterized by a Gaussian distribution with mean $1 + \alpha(S-1)\bar{f} - \lambda/r$ and a variance $1/(2Kr)$. For $r \gg \lambda$, the contribution from the exponential dominates the abundance distribution and the mean $\langle f\rangle$ can be approximated by $1 + \alpha(S-1)\bar{f}$. Due to self consistency ($\bar{f} = \langle f\rangle$), this yields $\langle f\rangle = 1/[1 - \alpha(S-1)]$, which is the steady state solution $f^* = N^*/K$ given by Eq. 2 of the main text.

S2.2 Mutualistic interactions can lift growth of a species above the critical growth rate

We saw that even for dispersal rates below λ_c, there can be a stable state with positive population sizes when the effect from positive interactions is strong enough. In the main

text we argue that in this case, mutualistic interactions increase the growth rate of a species, as given in Eq. 1 of the main text, so that the species' effective growth rate exceeds the minimal growth rate a hypothetical species would need in the absence of mutualistic interactions with other species to overcome noise-driven decay. To test this hypothesis, it is convenient to rewrite Eq. 1 of the main text in terms of some effective growth factor g_{eff}:

$$\partial_t N_{x,i}(t) = rN_{x,i}\left(g_{\text{eff}} - \frac{N_{x,i}}{K}\right) + \lambda(\bar{N}_i - N_{x,i}) + \sqrt{N_{x,i}}\,\eta_{x,i}\,, \tag{S14}$$

where we defined the new parameter $g_{\text{eff}} = 1 + \frac{\alpha}{K}\sum_{j,j\neq i}^{S} N_{x,j}$ as the effective growth factor. Using the mean-field solution discussed above, we can now derive the critical effective growth factor, $g_{\text{eff,c}}$, as a function of the dispersal rate (see black line in Fig S2). By definition, at $\lambda = \lambda_c$ we get $g_{\text{eff,c}} = 1$, since for dispersal rates below (above) λ_c a species goes extinct (survives) in the absence of mutualistic interaction, i.e. when $g_{\text{eff,c}} = 1$. Accordingly, for a species to subsist independently of other species for dispersal rates $\lambda < \lambda_c$, the effective growth factor would have to be larger than one; hence, $g_{\text{eff,c}} > 1$ in Fig S2. To probe our above hypothesis, we can use the mean field approach to directly calculate $\hat{N}_x$ and from there g_{eff} as $1 + \frac{\alpha}{K}(S-1)\hat{N}_x$. Indeed, for $\lambda > \lambda_t$, the the effective growth factor g_{eff} calculated from the positive stable solution for $\hat{N}_x$ [i.e. the upper branch in Fig 2*A*] lies above the critical value $g_{\text{eff,c}}$ (see red full circle in Fig S2). Hence, mutualistic interactions raise g_{eff} over the critical value. A similar argument can be made for interactions that increase the dispersal rate of species (see next section).

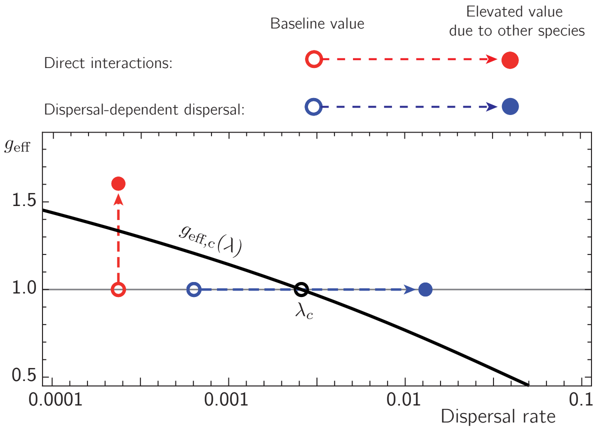

Fig S2. Species interactions can raise the species growth and dispersal parameters and thereby enable survival. A. Direct mutualistic interactions can raise the effective growth factor of species g_{eff} beyond the critical value, $g_{\text{eff,c}}$ (solid line), needed for survival. The red filled circle denotes the mean-field solution for g_{eff} in a metacommunity with mutualistic interactions as in Fig 2, for a dispersal rate just above λ_t ($S = 100$, $\alpha = 0.005$, $r = 0.3$, $K = 10$). For comparison, the open circle shows the g_{eff} when ignoring the effect of interactions (i.e. for an isolated species), which is one. **B.** Interactions between species that increase the species' dispersal rate, can raise the effective dispersal rate above the critical value λ_c. The blue filled circle denotes the mean-field solution for the effective dispersal rate in a metacommunity with density-dependent dispersal as in Fig 4, for a dispersal rate closely above λ_t ($S = 100$, $\beta = 0.02$, $r = 0.3$, $K = 10$). For comparison, the open circle shows the baseline dispersal rate λ when ignoring the effect of interactions (i.e. for an isolated species).

S2.3 Density-dependent dispersal

As detailed in the main text, we investigate density-dependent dispersal based on a dispersal rate given in Eq. 4 of the main text. Using the mean fields $\bar{f}_i$ and $\hat{f}_x$, the dynamics for every species on every patch can be written as

$$\begin{aligned} \partial_t f(t) =& f \frac{\partial \mathbf{F}}{\partial f} + \sqrt{f/K}\,\eta(t) \\ \text{with: } \mathbf{F} =& rf\left[1-\frac{f}{2}\right] \\ &+ \lambda(1+\beta K(S-1)\hat{f})\left[\bar{f}\log(f) - f\right] , \end{aligned} \tag{S15}$$

where, as in Eq. (S2), we omitted the species and patch index since all species and patches are assumed statistically identical. As discussed for direct mutualistic interactions, we can use this representation to derive the equilibrium probability distribution $\mathcal{P}[f]$ as a function of the mean fields:

$$\begin{aligned} \mathcal{P}[f,\bar{f},\hat{f}] =& \frac{1}{Z}\frac{1}{f^{1-2K\lambda(1+\beta k(S-1)\hat{f})\bar{f}}} \\ & \mathrm{e}^{2Krf\left[1-\frac{f}{2}\right]-2K\lambda(1+\beta k(S-1)\hat{f})f} , \end{aligned} \tag{S16}$$

where we omitted the dependence of $\mathcal{P}$ on r, K, α, and λ, and Z denotes the normalization constant. Imposing self-consistency, Eq. (S9), we can calculate $\bar{f}$ and $\hat{f}$ numerically and derive a closed solution for $\mathcal{P}[f]$. For sufficiently large S or β, the self-consistent solutions for $\bar{f}$ display a bifurcation depicted in Fig 4 including a discontinuous transitions and hysteresis upon varying the baseline dispersal rate λ. We comment, that at the critical dispersal rate λ_c, species interactions, also through dispersal, are negligible and thus λ_c for in the case of density-dependent dispersal is the same as for direct mutualistic interactions. The emergence of a stable solution with positive mean population size for dispersal rates below λ_c can be understood analogously to our results with direct mutualistic interactions (see above). Here, we argue that interactions between species can raise the effective dispersal rate of a species so that it exceeds the critical value λ_c. In detail, for $\lambda > \lambda_t$, the effective dispersal rate $\lambda(1+\beta(S-1)\hat{N})$ exceeds λ_c, whereas the baseline dispersal rate λ is below λ_c [see blue symbols in Fig S2].

We comment, that the emergence of a metacommunity-wide strong Allee effect for density-dependent dispersal even holds in the case of additional direct competitive interactions, when moderate. Specifically, Fig S3 displays the bifurcation of the mean population size based on the mean-field solution of a metacommunity following Eq. 1 of the main text with negative α (hence competitive interaction between species) and a density-dependent dispersal rate as in Eq. 4 in the main text. For increasing competition strength (i.e. increasing negative α), we see that the average population size decreases and the tipping point is shifted to larger baseline dispersal rates; however, the existence of a tipping point is preserved.

S3 Smoothening effect of stochastic fluctuations in metapopulations with global dispersal

One of our main results is that in the presence of mutualistic interactions between species, demographic noise can cause a sudden (discontinuous) transition between a regime where all species are extinct (inactive phase) and a regime of positive mean population size (active phase). Previous studies [14–16] have suggested that

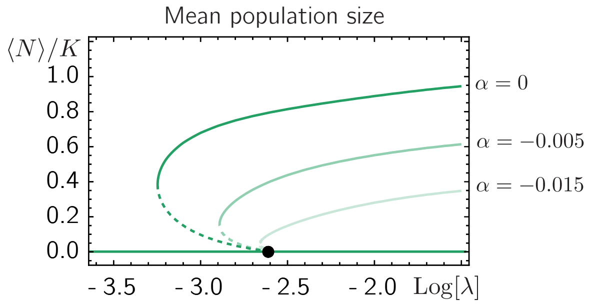

Fig S3. Moderate direct competition does not alter the emergence of a tipping point in a metacommunity with density-dependent dispersal. Based on our mean-field solution, additional direct competitive interactions with coefficient α (indicated on the right of panel) decrease the average population size and shift the tipping point to larger baseline dispersal rates λ. The full black circle indicates λ_c. Remaining parameters: $S = 100$, $\beta = 0.02$, $r = 0.3$, $K = 10$.

demographic fluctuations can also have the reverse effect, i.e. turn a discontinuous into a smooth transition. In their description, the population dynamics on a single patch shows bistability, with a stable fixed point at extinction and a second stable fixed point at a finite population size, separated by an unstable fixed point at intermediate population size [Allee threshold, compare Fig 1A]. In Ref. [14,16], the authors find that for short-range dispersal between patches (diffusive motion) demographic fluctuations can change the transition between an inactive and active phase from discontinuous to continuous when the dispersal rate between patches is low, or stochastic fluctuations are strong. In the following we investigate these findings for the case of global dispersal on the basis of our mean-field approximation and numerical simulations. To this end, we first describe the dynamics of a single population with size N that displays an Allee effect as

$$\partial_t N(t) = rN\left(\frac{N}{A} - 1\right)\left(1 - \frac{N}{K}\right) + \lambda(\bar{N} - N) + \sqrt{N}\eta\,, \tag{S17}$$

where we omitted the patch index to facilitate notation. This dynamics features bistability as illustrated in Fig 1A, with the Allee threshold (unstable fixed point) given by A. Analogous to Appendix Self-consistent mean-field approach, we employ a mean-field approximation, where the first term of the function $\mathbf{F}$ defined in Eq. (S2), which accounts for the population dynamics, is now given by $rf[1 + \frac{f}{2}(1 + \frac{K}{A}) - \frac{f^2 K}{A}]$. Fig S4A shows the obtained mean-field solutions for the mean population size $\langle N \rangle$ for $r = 0.3$, $K = 10$ and $A = 2$ and a varying dispersal rate λ (lines), together with respective numerical solutions (triangles and circles denote simulations starting at small and large initial mean population sizes, respectively). As expected, for very small dispersal rates, the population goes extinct, while for larger dispersal rates there is a stable solution with positive population size. Interestingly, for intermediate dispersal rates, there is a single stable solution, while for larger dispersal rates, the system either approaches extinction or a finite population size, depending on the initial population size. Our mean field approach reveals an unstable solution which separates these two stable outcomes. This strongly suggests that, depending on the dispersal rate, the metapopulation features a strong Allee effect (high dispersal rates) or not (intermediate dispersal rates). For a better comparison with the results of [14], who plotted the mean population size as a function of the linear growth rate, we will adapt our notation in the following to their description and investigate a possible change from an abrupt to a

smooth transition as they observed for short-range dispersal. Analogous to [14], we define the population dynamics

$$\partial f(t) = (af - bf^2 - cf^3) + \sqrt{f_x}\eta + \lambda(\bar{f} - f)\,, \tag{S18}$$

where, in contrast to [14], we included global dispersal (last term) instead of diffusive motion. η denotes Gaussian (white) noise with zero mean and a variance we set equal to 1 in the following for simplicity. For negative b and a and positive c, the deterministic dynamics of Eq. (S18) suggests bistability as illustrated in Fig 1A, characterizing an explicit strong Allee effect. Following [14], we now fix the parameters $b = -2$ and $c = 1$, and vary the parameter a for different dispersal rates λ [see Figure S4B]. Similar to what [14] observed for short range dispersal, when increasing a, we see that low dispersal rates promote a discontinuous transition from zero to finite mean population sizes, while for larger dispersal rate, the transition is smooth (i.e. continuous). Together with our results discussed above [Fig S4A], this strongly suggests that also for global dispersal, a population dynamics that features bistability in terms of a strong Allee effect, can, depending on the growth and dispersal parameters, result in a smooth transition between a regime of extinction and finite mean population sizes when embedded in a metacommunity undergoing demographic noise.

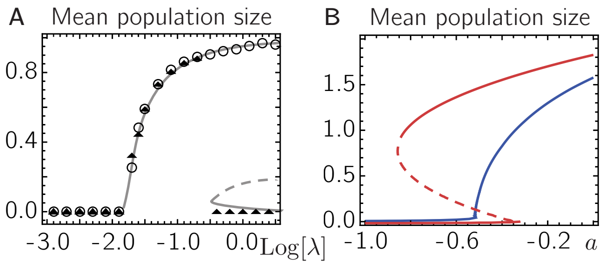

Fig S4. Smooth and discontinuous transitions in metapopulations with explicit strong Allee effect. A. Our mean-field approach (lines) and numerical solutions (circles and triangles correspond to simulations with initially large and small mean population size, respectively) of a metapopulation with explicit strong Allee effect, Eq. (S17), suggests bistability for large dispersal rates and a smooth transition from extinction to a finite population size for small dispersal rates. Remaining parameters: $K = 10$, $A = 2$, $r = 0.3$, for the numerical solutions: $P = 1000$. **B.** Similar to what [14] observed for short range migration, we find a change from a smooth transition for small dispersal rates (blue, $\lambda = 0.3$) to discontinuous transition with a regime of bistability for large dispersal rates (red, $\lambda = 3$). The dashed line denotes unstable solution.

S4 Metacommunities with random interactions

In our study of random interactions we consider the generalized metacommunity dynamics Eq. 3 (main text) with symmetric interactions (i.e. $\alpha_{j,i} = \alpha_{i,j}$) drawn from a Gaussian distribution with mean $\hat{\alpha}$ and standard deviation $\sigma/\sqrt{S}$. As explained in the main text, we focused on relatively small standard deviations σ, where previous studies suggest that, under well-mixed conditions, a community approaches a unique equilibrium state [17–19]. Specifically, [17, 18] state that for σ below $\sigma_c = \sqrt{2}(1 + \hat{\alpha})/(1 + \gamma)$, a well-mixed system with deterministic patch dynamics given the first line in Eq. 3 in the main text will settle into a unique stable equilibrium (here, γ denotes the symmetry of interactions with $\gamma = 1$ for symmetric and $\gamma = -1$ for

antisymmetric interactions; also, [17, 18] use different definitions for the mean and standard deviation of the random distribution). For the parameters used in Fig 3 and Fig S5, $\sigma_c \approx 0.7$, which lies above $\sigma = 0.5$; hence we expect the deterministic patch dynamics to be in the parameter regime with a unique stable state. While it is certainly interesting to study the dynamics Eq. 3 (main text) in the regime of multistability systems in a metacommunity context, here, we choose to avoid multistability due to large interaction difference to focus on the bistability due to demographic noise and mutualistic interactions alone.

For random interactions, we showed that the mean interactions of survivors, defined in the main text as $I_i := \langle(\alpha_{i,j}/K)N_{x,j}\rangle_i$, where the average is taken over all patches x and co-surviving species j at the last time point of our numerical solution, are positive in the intermediate dispersal rate regime of bistability and can be negative in for larger dispersal rates beyond the bistability regime [see Fig 3B]. While in Fig 3B, we joined all calculated mean interactions I_i from simulations with three different sets of random interactions, Fig S5A shows the distributions of I, $\mathcal{P}[I]$, plotted separately for each set of random interactions (each in a different color). As mentioned in the main text, in the regime where species survive, the number of species that go extinct and the final mean population size depend on the chosen set of random interactions and generally increase with the dispersal rate. Specifically, for the numerical solutions shown in Fig S5A, the number of surviving species for dispersal rates $\lambda = 10^{-2.8}$, $\lambda = 10^{-2.2}$, and $\lambda = 10^{-1.5}$ increases respectively from 22 to 32 to 44 for the set of random interactions that correspond to the solutions in blue, from 26 to 38 to 54 for the set of random interactions that correspond to the solutions in orange and from 21 to 39 to 53 for the set of random interactions that correspond to the solutions in green.

To investigate how the communities of remaining survivors of our numerical solution and their mean interaction compare to a mean-field approximation with a species-independent interaction coefficient, we plot the mean-field solution taking into account only the number of surviving species, S_{surv} and their average interaction coefficient $\hat{\alpha}_{\text{surv}} = \langle\alpha_{i,j}\rangle$ (where the average is taken only over surviving species i and j). Similar to Fig 2, we can now plot the bifurcation of the mean population size, i.e. the steady states of the metacommunity based on the mean-field solution with the parameters $S = S_{\text{surv}}$ and $\alpha = \hat{\alpha}_{\text{surv}}$ as a function of the dispersal rate λ. Interestingly, we observe that for dispersal rates in the bistability regime, the resulting bifurctations have tipping points λ_t^* that are close to the dispersal rate λ [see Fig S5B]. This suggests that random metacommunities self-organize to a state very close to the tipping point where they are very fragile against perturbations e.g. in the dispersal rate and species number.

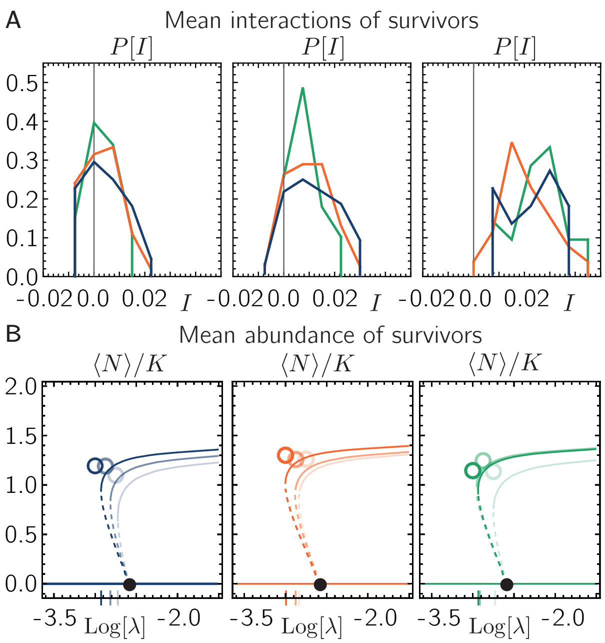

Fig S5. Bistability and self-organized tipping points in random metacommunities. A. Distributions $\mathcal{P}[I]$ for individual independent sets of random interactions as shown jointly in Fig 3*B* for $\lambda = 10^{-1.5}$, $\lambda = 10^{-2.2}$, and $\lambda = 10^{-2.8}$ (from left to right). Different colors correspond to the three different sets of interaction coefficients $\alpha_{i,j}$ used in in Fig 3*A* (drawn from a Gaussian distribution with mean $\hat{\alpha} = -0.01$ and standard deviation σ/S with $\sigma = 0.5$). **B.** Each panel shows the mean abundances of surviving species in our numerical solutions (open circles) for three different dispersal rates in the regime of bistability shown in Fig 3*A*. Dark to bright colors correspond to $\lambda = 10^{-3}$, $\lambda = 10^{-2.875}$, and $\lambda = 10^{-2.75}$, respectively; the three panels correspond to the three sets of random interaction coefficients used in A and Fig 3. Mean-field solution calculated based on the number of surviving species S_{surv} (ranging from ~ 25 at $\lambda = 10^{-3}$ to ~ 55 at $\lambda = 10^{-2.75}$) and mean of the interaction coefficients $\hat{\alpha}_{\text{surv}}$ for the corresponding three different dispersal rates (dark to bright colors correspond to $\lambda = 10^{-3}$, $\lambda = 10^{-2.875}$, and $\lambda = 10^{-2.75}$, respectively). The tipping point dispersal rates λ_t^* of the bifurcations are shown as ticks (dark to bright colors correspond to $\lambda = 10^{-3}$, $\lambda = 10^{-2.875}$, and $\lambda = 10^{-2.75}$, respectively) on the x-axis. Remaining parameters: $S = 100$, $K = 10$, $r = 0.3$, for the numerical solutions: $P = 500$.

References

1. Van Rossum G, Drake Jr FL. Python reference manual. Centrum voor Wiskunde en Informatica Amsterdam; 1995.
2. Wolfram Research. Mathematica, Version 12.3.1, Champaign, IL; 2021.
3. Gardiner CW, et al. Handbook of stochastic methods. vol. 3. springer Berlin; 1985.
4. Van Kampen NG. Stochastic processes in physics and chemistry. vol. 1. Elsevier; 1992.
5. Karlin S. A first course in stochastic processes. Academic press; 2014.
6. Iwasa Y. Free fitness that always increases in evolution. Journal of Theoretical Biology. 1988;135(3):265–281.
7. Barton NH, De Vladar HP. Statistical mechanics and the evolution of polygenic quantitative traits. Genetics. 2009;181(3):997–1011.
8. Nachman G. Effects of demographic parameters on metapopulation size and persistence: an analytical stochastic model. Oikos. 2000;91(1):51–65.
9. Eriksson A, Elías-Wolff F, Mehlig B. Metapopulation dynamics on the brink of extinction. Theoretical population biology. 2013;83:101–122.
10. Casagrandi R, Gatto M. A persistence criterion for metapopulations. Theoretical population biology. 2002;61(2):115–125.
11. Fisher RA, Corbet AS, Williams CB. The Relation Between the Number of Species and the Number of Individuals in a Random Sample of an Animal Population. The Journal of Animal Ecology. 1943;12(1):42.
12. Pielou EC, et al. An introduction to mathematical ecology. An introduction to mathematical ecology. 1969;.
13. Patil GP, Pielou EC, Waters WE, Waters W, Waters WA. Statistical ecology: spatial patterns and statistical distributions. vol. 1. Penn State University Press; 1971.
14. Villa Martín P, Bonachela JA, Levin SA, Muñoz MA. Eluding catastrophic shifts. Proceedings of the National Academy of Sciences. 2015;112(15):E1828–E1836.
15. Sardanyés J, Piñero J, Solé R. Habitat loss-induced tipping points in metapopulations with facilitation. Population Ecology. 2019;61(4):436–449.
16. Weissmann H, Shnerb NM. Stochastic desertification. EPL (Europhysics Letters). 2014;106(2):28004.
17. Bunin G. Ecological communities with Lotka-Volterra dynamics. Physical Review E. 2017;95(4):1–8.
18. Biroli G, Bunin G, Cammarota C. Marginally stable equilibria in critical ecosystems. New Journal of Physics. 2018;20(8).
19. Galla T. Dynamically evolved community size and stability of random Lotka-Volterra ecosystems(a). Epl. 2018;123(4):1–13.
